# Identification of dominating factors affecting vadose zone vulnerability by a simulation method

**DOI:** 10.1038/srep45955

**Published:** 2017-04-07

**Authors:** Juan Li, Beidou Xi, Wutian Cai, Yang Yang, Yongfeng Jia, Xiang Li, Yonggao Lv, Ningqing Lv, Huan Huan, Jinjin Yang

**Affiliations:** 1 College of Water Sciences, Beijing Normal University, Beijing 100875, China; 2 State Environmental Protection Key Laboratory of Simulation and Control of Groundwater Pollution, Chinese Research Academy of Environmental Sciences, Beijing 100012, China; 3 Center for Hydrogeology and Environmental Geology Survey, China Geological Survey, Baoding 071051, China; 4 School of Environment, Beijing Normal University, Beijing 100875, China

## Abstract

The characteristics of vadose zone vulnerability dominating factors (VDFs) are closely related to the migration and transformation mechanisms of contaminants in the vadose zone, which directly affect the state of the contaminants percolating to the groundwater. This study analyzes the hydrogeological profile of the pore water regions in the vadose zone, and conceptualizes the vadose zone as single lithologic, double lithologic, or multi lithologic. To accurately determine how the location of the pollution source influences the groundwater, we classify the permeabilities (thicknesses) of different media into clay-layer and non-clay-layer permeabilities (thicknesses), and introduce the maximum pollution thickness. Meanwhile, the physicochemical reactions of the contaminants in the vadose zone are represented by the soil adsorption and soil degradability. The VDFs are determined from the factors and parameters in groundwater vulnerability assessment. The VDFs are identified and sequenced in simulations and a sensitivity analysis. When applied to three polluted sites in China, the method improved the weighting of factors in groundwater vulnerability assessment, and increased the reliability of predicting groundwater vulnerability to contaminants.

China has 5118 groundwater wells in 202 cities^1^. In more than 60% of these wells, the groundwater quality exceeds Class III (unfit for human potable water) in the *Quality standard for ground water* assessment^2^. The main components failing the standard are Total Hardness, Total Dissolved Solids (TDSs), Fe, Mn, N, F^−^, and SO_4_^2−^. Ar, Pb, Cr^6+^, and Cd have also been detected in some wells. In 2015, the China State Council issued its *Action Plan for Prevention and Control of Water Pollution*, which highlighted the prevention and control of groundwater pollution as a major problem in China. However, the hydrogeological characteristics (such as the geology and physiognomy) of China’s land regions are quite variable. Therefore, when assessing the groundwater pollution intensity (GPI) in China, we combine the pollution characteristics and vadose zone characteristics^3^. Thus, the GPI assessment includes the pollution sources hazards (PSH) and the groundwater intrinsic vulnerability (GIV). The GIV assessment is graded by the DRTAS^3^,^4^ model, which improves the DRASTIC^5^ model for unconfined aquifers. However, the DRTAS weight index does not account for the structure and characteristics variable of the vadose zone. To address this deficiency, we identify and sequence the vadose zone vulnerability dominating factors (VDFs), and weight them for GIV assessment.

The VDF weights are important for GIV assessment^6^,^7^,^8^, which only considers the hydrogeological factors^9^, because the vadose zone controls the migration and transformation of contaminants^10^. The mechanisms controlling the water flow and contaminant transport through the vadose zone have been extensively investigated for several decades. Studies have ranged from small-scale laboratory experiments^11^,^12^,^13^,^14^ to large experimental field operations^15^,^16^,^17^,^18^,^19^. For instance, the media type of the vadose zone determines the pollutant damping from the pedosphere bottom to the groundwater table. Within the vadose zone, pollutants may be biodegraded, mechanically filtered, chemically reacted, volatilized and dispersed. Biodegradation and volatilization usually reduce with increasing depth. The penetrating paths and lengths are controlled by the media, whereas the hydraulic conductivity is mainly influenced by pore diameter and fracture. When the hydraulic conductivity is higher, the contaminants more easily migrate into the groundwater, and the vadose zone becomes more vulnerable. Therefore, the influence of VDFs on contaminant migration should be analyzed in terms of the groundwater specific vulnerability (GSV), which considers particular pollution sources or human activities with GIV assessment^9^. Although the GSV of pesticides^20^ and nitrogen^21^,^22^,^23^ has been reported, the applicability of this measure to wider regions has not been adequately tested.

GSV is usually assessed by statistical modeling and simulation^24^. The simulation model for analyzing the VDFs must be properly selected. The one-dimensional finite-element model HYDRUS-1D, which simulates the movement of water, heat, and multiple solutes in variably saturated heterogeneous or layered soils under various atmospheric and other boundary conditions, estimates the fate and transport of contaminants in vadose zones^25^, is better for VDFs analysis. HYDRUS-1D has simulated the transport of agricultural chemicals^26^, heavy metals, and organic pollutants^27^.

Therefore, the present paper simulates the fate and transport of contaminants in the vadose zone by HYDRUS-1D, and sequences the influence of VDFs on the vadose zone vulnerability. By applying the VDFs rankings as factor weights, we can improve the GSV assessment, and provide support for groundwater pollution control and protection. The study process is shown in Fig. 1. We first identify the groundwater pollution characteristics and the pore-phreatic hydrogeological conditions in China. Second, we generalize the vadose zone structure and identify and express the VDFs as a series of parameters. Finally, we establish a VDF sequencing method for specific areas, thus providing a GSV assessment.

## Method

### Analysis of groundwater pollution characteristics in China

**Figure 2:**
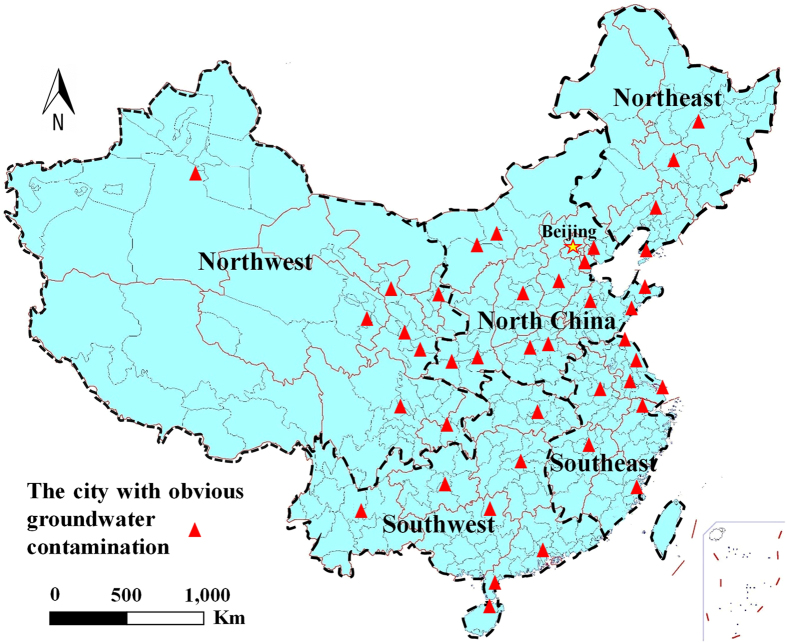
Locations with obvious groundwater pollution in China (Map generated using ArcGIS 10.0, http://www.esri.com, and the Microsoft Office 2010 software for Windows 2003/XP/VISTA/7).

The characteristics of groundwater pollution differ among the various regions of China depend on 5118 groundwater monitoring wells’ location and groundwater quality. In the northeastern parts, the main contaminants are nitrogen, volatile phenols, and petroleum pollutants, which are often released by heavy industry and oil exploration^28^,^29^,^30^. In Northern China, the main contaminants exceeding the standards are N, Fe, Mn, and petroleum pollutants^31^,^32^,^33^,^34^,^35^, whereas pollutants in northwestern China are dominated by N, Cr, and Pb^36^,^37^. The groundwater is generally high-quality in the southwestern parts, but is sometimes excessively contaminated by Fe, Mn, and volatile phenols^38^,^39^. In southeast China, unconfined groundwater contaminants are commonly found around the Yangtze River Delta^40^ and the Pearl River Delta^41^. The main contaminants exceeding the standards are N, Hg, Cr, and Mn^42^,^43^. General contaminants include nitrates, heavy metals, and organic matters. Sites with obvious groundwater pollution in China are indicated in Fig. 2. According to the National Base of Groundwater Environment Survey Assessment (2012–2015) compiled by the Ministry of Environmental Protection of China and the National Evaluation of Groundwater Pollution Survey (2005–2015) compiled by the Chinese Geological Bureau, groundwater contaminants in China have concentrated in unconfined aquifers over the past 10 years. Given these characteristics, we target pore-phreatic aquifers for protection in the present study.

### Analysis of pore-phreatic hydrogeological conditions

China can be divided into six hydrogeological regions with different climate zones, geologies, and geomorphological conditions^44^ (I) a sub-humid hydrogeological region in the north and northeast, (II) a semi-arid hydrogeological regions encompassing Inner Mongolia and the Loess Plateau, (III) an arid hydrogeological region of inland basin in the northwest, (IV) a humid hydrogeological region in the south, (V) coastal subtropical and tropical hydrogeological regions, and (VI) a cold, arid hydrogeological region on the Qinghai–Tibet Plateau.

#### Conceptualization of vadose zone structure

Based on the hydrogeological condition of the pore-phreatic aquifer in China and the permeability coefficients of the various media^45^,^46^, the vadose zone can be separated into single lithologic, double lithologic, and multi lithologic types. The empirical permeability coefficients in different media are listed in Table 1.

#### Single lithologic type

**Figure 3:**
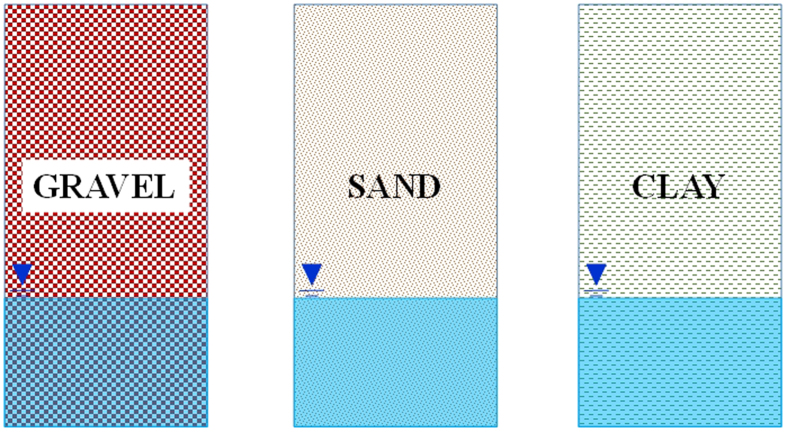
Single lithologic type of vadose zone structure (generated by Microsoft Office 2010 software for Windows 2003/XP/VISTA/7).

The single lithologic type is composed of a single porous medium such as sand, silt, or clay (Fig. 3). The groundwater is stored in the same porous media. The changing permeability coefficient reflects the lithologic structure variable^47^.

#### Double lithologic type

**Figure 4:**
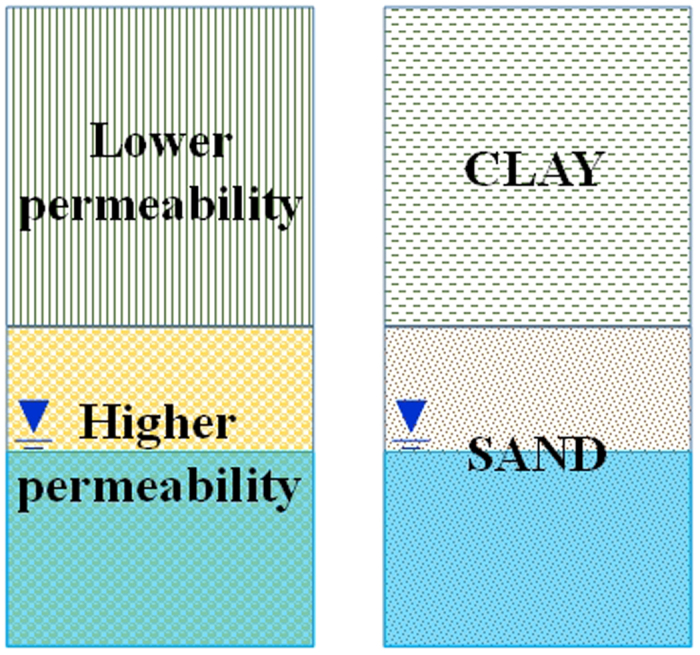
Double lithologic type of vadose zone structure (generated by Microsoft Office 2010 software for Windows 2003/XP/VISTA/7).

The double lithologic type consists of an upper medium with lower permeability and a lower medium with higher permeability (Fig. 4). The groundwater resides in the lower medium. The upper medium may be clay or silt, whereas the lower media may be sand or gravel. The permeability coefficients of the two layers differ by more than two orders of magnitude at least.

#### Multi lithologic type

**Figure 5:**
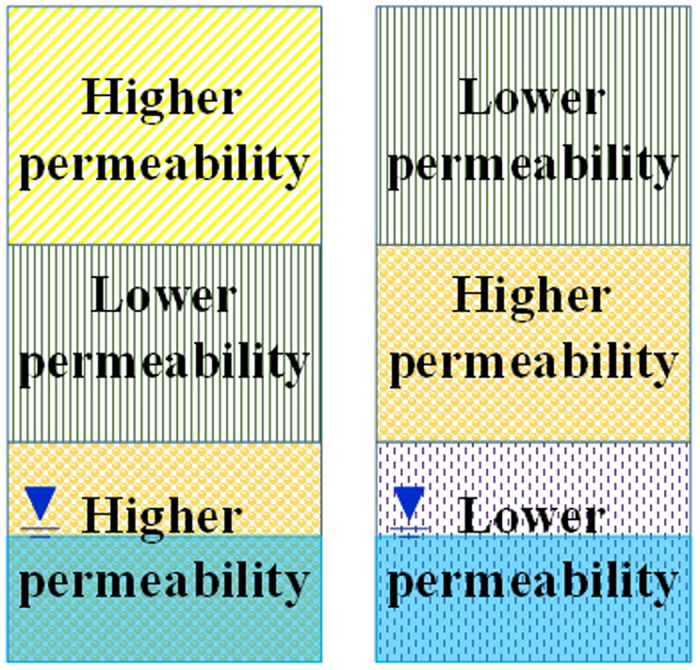
Multi lithologic type of vadose zone structure (generated by Microsoft Office 2010 software for Windows 2003/XP/VISTA/7).

The multi lithologic type consists of two or more pore media with different permeabilities. The vadose zone consists of three or more layers of materials such as silty clay, fine sand, and clay (Fig. 5). The permeability coefficients of the different layers differ by more than one order of magnitude.

#### Identification of VDFs

The VDFs should be easily obtained and quantifiable. In this study, they are identified by groundwater vulnerability assessment.

**Figure 6:**
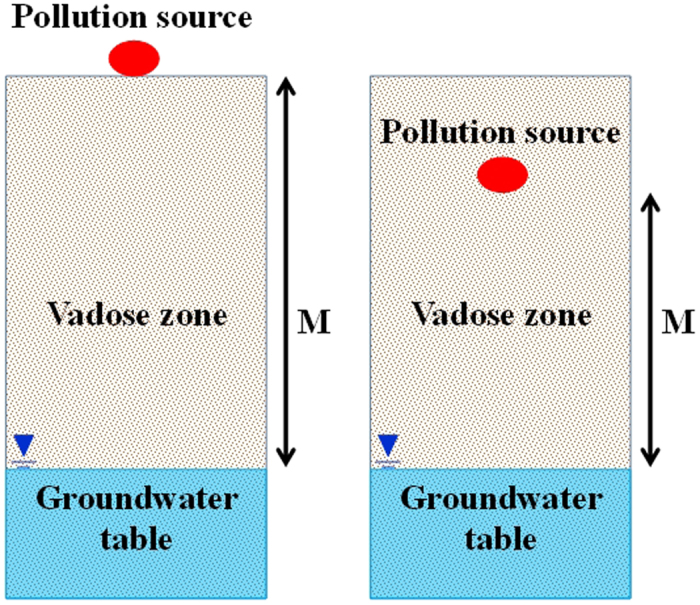
Conceptual diagram of maximum pollution thickness (M) (generated by Microsoft Office 2010 software for Windows 2003/XP/VISTA/7).

The most popular model for assessing groundwater intrinsic vulnerability is the DRASTIC model^5^ developed by a committee of the United States Environmental Protection Agency (EPA). However, as DRASTIC operates on regional scales, some of the DRASTIC factors are unsuitable for assessing vadose zone vulnerability and protecting unconfined groundwater at the site scale. Therefore, we must select the factors that most influence the vadose zone vulnerability^4^. The vadose zone is most closely associated with the groundwater level (D) and impact of the vadose zone (I), where D represents the distance from the ground surface to the groundwater table. In practice, the pollution sources occupy both the ground surface and the vadose zone. The further the pollution source from the groundwater table, the longer the time of reaction between the contaminants and the soil media. Consequently, the reaction will be more sufficient, and the vadose zone will be less vulnerable. In this paper, we replace D by the maximum pollution thickness (M), which defines the distance between the pollution sources and groundwater table, and which more accurately reflects the influence of the pollution source on the groundwater (Fig. 6).

The factor I represents the impact of the vadose zone, which depends on the media types and thicknesses. The permeability of a vadose medium controls the transport velocity of the pore water, and is influenced by the pore number and grain size. More specifically, vadose-medium permeability can be separated into clay layer permeability (K_1_) and non-clay layer permeability (K_2_). Noting that the vadose antifouling performance is higher in thicker media, we also introduce two thickness parameters; the clay layer thickness (M_1_) and the non-clay layer thickness (M_2_). The migration times and concentrations of the groundwater contaminants are also affected by the convection, dispersion, adsorption and biodegradation of the contaminants in the vadose zone. The pollution sources and contaminants should be factored into the groundwater specific vulnerability. Considering the adsorptions and degradations of different contaminants in the vadose zone, and modeling the chemical reactions in HYDRUS-1D, we introduce two VDFs called the soil adsorption (Kd) and soil degradability (μ). Soil degradability includes the biodegradation, mechanical filtration, chemical reaction, volatilization, and dispersion of aqueous-phase contaminants in the vadose zone.

In conclusion, the VDFs include the maximum pollution thickness (M), the clay and non-clay layer thicknesses (M_1_ and M_2_ respectively), the clay and non-clay layer permeabilities (K_1_ and K_2_ respectively), the soil adsorption (Kd), and the soil degradability (μ).

#### VDFs of three typical vadose zone structures

Recall that the single lithologic type comprises one lithologic porous medium. As the VDFs for this type, we select M, K_1_ or K_2_, Kd, and μ, and refer to K_1_ or K_2_ simply as the soil permeability (K).

In the double lithologic type, the permeability is higher in the lower medium than in the upper medium. Hence, the VDFs should account for the different media types. For this purpose, we selected M, M_1_, K_1_, K_2_, Kd, and μ as the VDFs. These factors mainly reflect the dominant role of the clay layer in the contaminants’ migration. The values of Kd and μ were those of the layer with the lower adsorption and degradation determined in HYDRUS-1D.

The multi lithologic type consists of two or more pore media with different permeabilities, and the vadose zone consists of three or more layers. To reflect this composition, we selected M, M_1_, K_1_, K_2_, Kd, and μ as the VDFs. The values of Kd and μ were those of the layer with lower adsorption and degradation determined by HYDRUS-1D. The equivalent permeability of the clay or non-clay layer is calculated by Eq. (1).


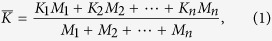


where 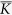 is the equivalent permeability coefficient, (cm/s);

*K*_1_, *K*_2_, …, *K*_*n*_ are permeability coefficients (cm/s);

*M*_1_, *M*_2_, …, *M*_*n*_ are the thicknesses of the layers (m).

The VDFs of the different vadose structures are summarized in Table 2.

#### Sequence of the VDFs

Using the identified VDFs, we simulated the contaminants’ concentrations and times of migration to unconfined groundwater at the site scale by HYDRUS-1D, and weighted the VDFs for assessing the GSV assessment. The VDFs were sequenced by the following method, which includes three steps:

Step 1: Collect the contaminants and hydrogeological data in the target case. The pollution source data include the source location (ground surface or underground) for determining the maximum pollution thickness (M), the source release mode (indirect or continuous) for determining the HYDRUS-1D boundary conditions, the source leakage amounts and initial contaminant concentrations for determining the initial conditions in HYDRUS-1D, and the reaction type of the contaminants with the vadose zone.

The hydrogeological data include the annual precipitation for determining the HYDRUS-1D boundary conditions, the geological profile information to conceptualize the vadose zone structure and obtain the permeability coefficients of the different media, the groundwater depth for determining the maximum pollution thickness (M), and the contaminant concentrations in the groundwater for checking the simulation results.

Step 2: Conceptualize the vadose zone structure using the hydrogeological data. Identify the VDFs relevant to this structure, and calculate the VDF parameters using the measured or literature values at each site.

Step 3: Sequence the VDFs through a sensitivity analysis. Establish the site-specific HYDRUS-1D model based on the contaminants and hydrogeological data. The simulation process involves building the model structure, handling the sinks and sources, determining the boundary conditions, selecting the parameters, and determining the simulation time.

The governing equations in HYDRUS-1D are the water flow equation (Eq. 2) and the solute transport equation (Eq. 3).


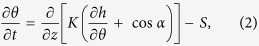


where *h* is the water pressure head [L], *θ* is the volumetric water content [L^3^L^−3^], *t* is time [T], *z* is the spatial coordinate [L] (positive upward), *S* is the sink term [L^3^L^−3^T^−1^], and *α* is the angle between the flow direction and the vertical axis (i.e., *α* = 0° for vertical flow, 90° for horizontal flow, and 0° < α < 90° for inclined flow). *K* is the unsaturated hydraulic conductivity function [LT^−1^] given by *K(h, x*) = *Ks(x) Kr(h, x*), where *Kr* [−] and *Ks* [LT^−1^] denote the relative and saturated hydraulic conductivities, respectively. In the transport equation


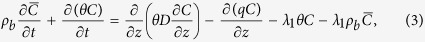


*C* is the solute concentration in the liquid [ML^−3^], and 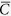 is the solute concentration adsorbed to the medium particles [ML^−3^]. *θ* is the volumetric water content [L^3^L^−3^], *ρ*_*b*_ is the soil bulk density [M L^−3^], *D* is the dispersion coefficient [L^2^T^−1^] of the liquid phase, *q* is the volumetric flux density [LT^−1^], and *λ*_*1*_ and *λ*_*2*_ are the first-order rate constants of the solutes [T^−1^].

To capture the influence of the VDFs, we take two ratios of the simulation results as reference indices. The first reference is the ratio *C*_*max*_/*C*_*0*_, where *C*_*max*_ and *C*_0_ are the maximum contaminant concentration at the groundwater table and the initial contaminant concentration at the pollution source, respectively. The second ratio is *t*/*T*, where *t* is the time at which the contaminant concentration first reaches *C*_*max*_, and *T* is the total simulation time. A higher *C*_*max*_/*C*_*0*_ implies a higher vadose vulnerability, and easier contamination of the groundwater. A higher *t*/*T* implies a lower vadose vulnerability, and higher resistance to groundwater contamination. To unify these two effects on the groundwater, we define the vadose zone vulnerability index *n* as follows:


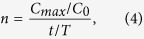


A high *n* implies high vadose vulnerability and easy contamination of the groundwater. In the HYDRUS-1D simulations, *C*_*max*_ and *C*_*0*_ are specified in mg/L, and *t* and *T* are expressed in days.

The VDFs are sequenced in a sensitivity analysis, which changes the value of a target factor while maintaining other factors constant^48^. The effect to which the target factor influences the result is then observed. In the present analysis, we increased or decreased each VDF value by 20%^49^,^50^, maintaining the other values constant, and calculated the vadose zone vulnerability.

The vulnerability index *n* of the vadose zone reflects the relationship between the VDF variables and the variable of the vadose zone vulnerability. To highlight the effect degree of *n* with each VDF, we computed the vulnerability index amplitude *δ* as follows:


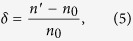


where *n*′ is the vulnerability index with a single VDF variable, and *n*_*0*_ is the initial vulnerability index.

The average absolute value |*δ*| of the vulnerability index amplitude is then calculated as


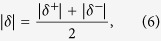


where |*δ*^+^| and |*δ*^−^| are the absolute amplitudes of the vulnerability index when the single VDF is increased and decreased by 20%, respectively. Finally, the VDFs are ranked by their |*δ*| values.

### Case study

To date, comprehensive correlation analyses between pollution sources and groundwater pollution degree have been rarely conducted in China^3^. Based on the characteristics of China’s groundwater pollution and the conceptualized vadose zone structure in pore-phreatic water, we selected three sites for the case study; a chromium slag dump with a single lithologic structure, a non-standard landfill with ammonia nitrogen and a double lithologic structure, and a sewerage leach pit with chlorobenzene and a multi lithologic structure.

**Figure 7:**
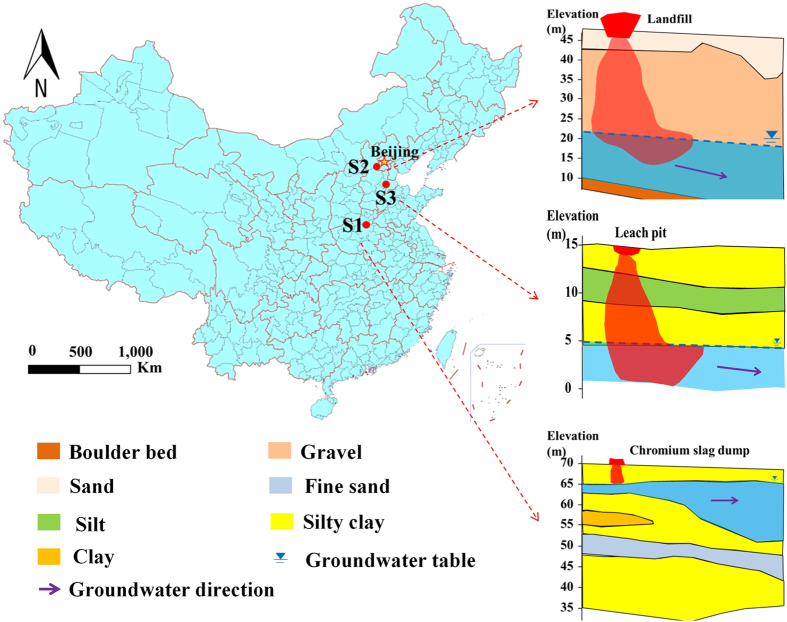
Location of case study sites (Map generated using ArcGIS 10.0, http://www.esri.com, and the Microsoft Office 2010 software for Windows 2003/XP/VISTA/7).

The three sites are located in the North China Plain, a sub-humid hydrogeological region. The elevation is typically below 100 m and the terrain is smooth. The main geomorphic types are foreland pluvial fan, alluvial plain, and coastal region. There are multiple aquifers and a complex stratigraphic structure. The aquifer lithology is dominated by fine sand and fine silt. The groundwater at 60–80 meters below the Earth’s surface is mainly unconfined or micro-confined groundwater^51^. The site locations and their vadose zone structures are shown in Fig. 7.

**Figure 8:**
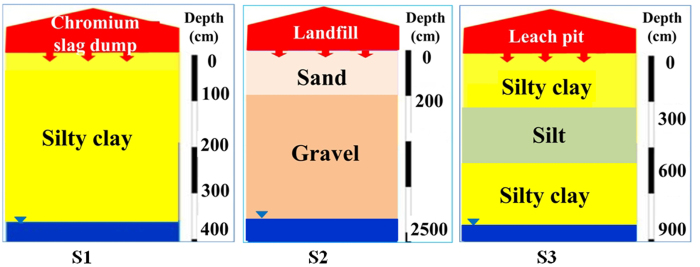
Conceptualized vadose zone structures of the sites in the case study (generated by Microsoft Office 2010 software for Windows 2003/XP/VISTA/7).

The chromium slag dump (Site 1) occupies a chromium industry founded in the 1980s. Chromium slags are stored in the open environment with no anti-seepage measures. The leachate directly enters the vadose zone through eluviation. The vadose zone is a 4 m-thick layer of silty clay, with a permeability coefficient of 3.3 × 10^−5 ^cm/s. The annual average groundwater table is approximately 4 m. Based on the available information, the vadose zone structure is classified as single lithologic type (Fig. 8). The chromium concentration was 400 mg/L in the leachate (recorded in the leachate monitoring results) and 55 mg/mL in the groundwater monitoring well, which is located 20 m downgradient of the dump. Therefore, the dump poses serious threats to the groundwater.

The non-standard landfill (Site 2) with no anti-seepage was founded in the 1990s. The leachate directly enters the vadose zone through eluviation. The vadose zone structure comprises a 4–8 m-thick layer of medium sand with a permeability coefficient of 1.2 × 10^−2 ^cm/s, and an 18–22 m-thick gravel layer with a permeability coefficient of 4.0 × 10^−2 ^cm/s, placing it in the double lithologic category (Fig. 8). The groundwater table is approximately 25 m. The ammonia nitrogen concentration was 1810 mg/L in the landfill leachate (2011 monitoring results), and 63.9 mg/mL in the groundwater monitoring well, which is located 30 m downgradient of the landfill.

The sewage leach pit (Site 3) occupies a chemical plant founded in the 2000s. The sewage directly enters the vadose zone, which comprises a 2.5 m-thick silty clay layer, a 3 m-thick silt layer, a 3.5 m-thick silty clay layer, and a 4 m-thick silty sand layer. Thus, the vadose zone is classified as multi lithologic (Fig. 8). The permeability coefficients of the 2.5-m, 3.0-m, 3.5-m, and 4.0-m layers are 3.5 × 10^−5^, 7.5 × 10^−5^, 3.5 × 10^−5^ and 3.0 × 10^−4 ^cm/s, respectively, and the groundwater table is approximately 9 m. The chlorobenzene concentration was 138 mg/L in the leach pit (2011 monitoring results), and 5 mg/mL in the groundwater monitoring well, which is located 5 m downgradient of the leach pit.

The VDFs of each site were identified and sequenced under the site conditions. The analysis proceeded through pollution source identification, hydrogeological conditions analysis, numerical setup and calculations, and VDF sequencing.

## Results and Discussion

### Pollution source identification

The contaminants in the pollution source data of each site are presented in Table 3. The source locations, release and leakage amounts of the sources, initial contaminant concentrations, and the environmental behaviors of the contaminants in the vadose zones, were input to the simulation.

### Hydrogeological condition analysis

Table 4 lists the hydrogeological data of the sites, including the annual precipitation, the conceptualized vadose zone structure, the VDFs, the groundwater depth, and the groundwater concentrations of the contaminants.

### Simulation setup and computation

The soil hydraulic parameters, solute transport parameters, and solute reaction parameters were set in the HYDRUS-1D model. The parameters, including the residual and saturated soil water contents (*θ*_*r*_ and *θ*_*s*_ respectively), the parameters in the soil water retention function (*α, n*, and *L*), the longitudinal dispersivity in the soil (*D*_*L*_), the diffusion coefficient in free water (*D*_*w*_), and the adsorption isotherm and degradation coefficients (Kd and μ respectively)^26^, are shown in Table 5. The values of *θ*_*r*_, *θ*_*s*_ *α, n*, and *L* were set to their default values in HYDRUS-1D, whereas Ks, bulk density, and Kd were measured. The values of dispersivity, diffusion and μ were determined from the literature^52^,^53^,^54^.

The initial soil concentration of the contaminants was set to zero at all three sites. The initial pressure head over the whole vadose zone profile was set from −100 cm at the surface to 0 cm at the groundwater table. The upper flow boundary condition was assumed as constant flux. Based on the annual precipitation, which is assumed to infiltrate the vadose zone, the upper flux was set to 2.5 cm/d at site 1, and 3 cm/d at sites 2 and 3. The lower flow boundary condition was assumed as free drainage. A concentration flux boundary condition was imposed on the upper solute, and the contaminant concentration was set to 400 mg/L at site 1, 1810 mg/L at site 2, and 138 mg/L at site 3, reflecting the actual conditions. The lower solute was subjected to a zero-concentration boundary condition. The simulation time was set to the existence time of the site (30 years at site 1, 20 years at site 2, and 15 years at site 3). The boundary conditions, parameters and source/sink terms were input to HYDRUS-1D. The vadose zone vulnerability index *n* was calculated by Eq. (4), using the calculated values of *C*_*max*_ and *t*. The results are shown in Table 6.

Comparing the monitored groundwater concentrations of the contaminants near the pollution sources (55 mg/L of chromium at site1, 63.9 mg/L of ammonia nitrogen at site 2, and 5 mg/L of chlorobenzene at site 3) with the *C*_*max*_ values in Table 6, we find that the simulated values are larger than the monitored values. This discrepancy is attributed to the parameters being determined partially from measured values and partially from empirical values provided in the literature, which will certainly differ under the actual site conditions. For instance, the vadose zone structure was generalized from a geological section map, which neglected some of the soil media, and the groundwater wells are a certain distance from the seepage point. In general, the simulated contaminant concentrations at the three sites were close to their monitored values, and the simulations captured the migration and transformation of contaminants in the vadose zone. Therefore, the simulation results are adequate for ranking the VDFs.

### Ranking of the VDFs

Based on the analysis results in Tables 4 and 5, the values of the VDFs for each site are shown in Table 7.

Each VDF was increased or decreased by 20%, retaining the other VDFs constant, and the vadose zone vulnerability was calculated. The vulnerability index amplitude *δ* was then calculated by Eq. (5), and the average absolute amplitude of the vulnerability |*δ*| was determined by Eq. (6). The results of site 1, 2, and 3 are presented in Tables 8,9 and 10, respectively.

Based on the |δ| values, the VDF parameters for chromium at site 1 are ranked as M, Kd, μ, K; for ammonia nitrogen at site 2 they are ranked as M, μ, Kd, M1, K1, K2; for chlorobenzene at site 3 they are ranked as M, μ, Kd, K1, K2, M1.

### Application of the VDFs

**Figure 9:**
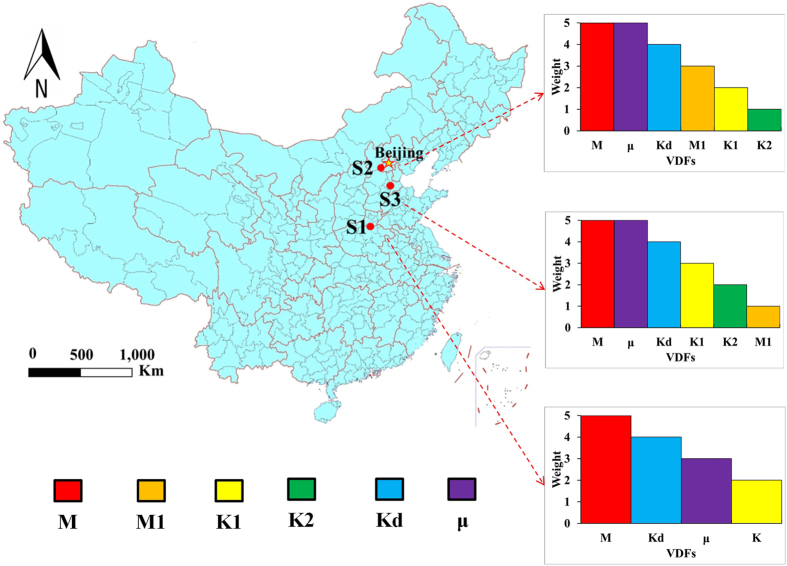
VDF weights at the three study sites (Map generated using ArcGIS 10.0, http://www.esri.com, and the Microsoft Office 2010 software for Windows 2003/XP/VISTA/7).

As shown in the results, the VDF rankings depend on the contaminants and hydrogeological conditions. After referencing the weightings of the DRASTIC model, the greatest and smallest impact factors are 5 and 1, respectively^6^. This referencing converts the rank results to VDF weights (Fig. 9), which can be used for groundwater specific vulnerability; for instance, for assessing groundwater pollution intensities^3^. Research at typical sites will provide references for VDF analysis at other sites with similar hydrogeological conditions.

### Method’s applicability

Evaluating the effects of hydrogeological conditions on contaminant migration is essential for assessing groundwater specific vulnerability. Moreover, identifying the main VDFs from the hydrogeological conditions can improve the assessment accuracy. For sequencing the VDFs, we simulated the contaminants in the vadose zone and conducted a sensitivity analysis on the VDFs. This approach is more objective than the overlay and index method, thereby increasing the rationality and practicality of predicting the groundwater specific vulnerability to contaminants. Because it combines the contaminants with VDF variables, the method is suitable for small-scale groundwater pollution sources. In addition, the VDFs and conceptualized vadose zone structures are applicable to pore water.

The method was applied at three sites with different contaminants and vadose zone structures. The VDF rankings were closely related to the physicochemical properties of the contaminants. At sites 1, 2 and 3, the vadose zone vulnerability was mainly influenced by the soil adsorption of chromium, the nitrification/denitrification of ammonia nitrogen in the soil, and the soil absorption of chlorobenzene, respectively. The simulation and ranking of the VDFs results revealed not only the contamination degree of the vadose zone, but also the VDFs that most significantly affected the contaminant behaviors. Combined with assessments of groundwater pollution intensity^3^, this method could facilitate the adequate protection of unconfined groundwater. Future study should extend the method to other contaminants in typical vadose zone structures, and explore a wider range of regions.

Conceptualizing the vadose zone structure is suitable not only for pore water, but also for fracture water and karst water. The vadose zones of fracture water are broadly classified as weathering and structural fracture types. Weathering fracture types includes a strongly weathered zone at the top and a weakly weathered zone at the bottom. Similarly to the double lithologic type, the groundwater is generally stored in the weakly weathered zone. Structural fracture types show little or no change throughout the vadose zone, and the groundwater is generally stored in the fractures, resembling the single lithologic type. The vadose zones of karst water are classifiable as bare karst, covered karst, and karst-hole. The bare karst type is similar to the structural fracture type, in that groundwater is generally stored in the corrosion fractures. The covered karst type includes low-permeability clay layers at the top and corrosion fractures with higher permeability at the bottom. Again, groundwater is generally stored in the corrosion fractures, but this type resembles the double lithologic type. The karst-hole type is characterized by well-developed corrosion fractures. The groundwater is freely recharged by surface water and stored in an underground river system. Therefore, the karst hole is a preferential migration passage with no influence on contaminant transport. The VDFs of fracture water and karst water could be analyzed by the pore water method, and their data ranges determined. In this way, the sequence VDF method can be extended to more regions.

In summary, the sequence VDF method combined with simulations of the groundwater specific vulnerability will increase the reliability of predicting groundwater specific vulnerability. The method is flexible, as VDFs and parameters can be added or removed according to the hydrogeological and hydrological statuses of specific areas.

## Conclusions

We established a method for identifying and sequencing VDFs. The method introduces the vadose zone vulnerability index *n* and the vulnerability index amplitude *δ* for quantifying the contribution degree of each VDF. The sensitivities of the VDFs depend on the contaminants, and the VDFs can be ranked for assessing groundwater specific vulnerability. In the unconfined aquifers of China, the main groundwater specific contaminants are nitrates, heavy metals, and organic matters. Therefore, we sequenced the VDFs at a single lithologic site contaminated with chromium (site 1), a double lithologic site contaminated with ammonia nitrogen (site 2), and a multi lithologic site contaminated with chlorobenzene (site 3).The identified VDFs were the maximum pollution thickness (M), clay layer thickness (M_1_), non-clay layer thickness (M_2_), clay layer permeability (K_1_), non-clay layer permeability (K_2_), soil adsorption (Kd), and soil degradability (μ). In order of decreasing influence, the VDFs at each site were ranked as M, Kd, μ, K at site 1; M, μ, Kd, M_1_, K_1_, K_2_ at site 2; and M, μ, Kd, K_1_, K_2_, M_1_ at site 3. From the VDF ranking results, we can assess the groundwater pollution intensity^3^.

The type of vadose zone structure (single lithologic, double lithologic or multi lithologic) can be determined by analyzing the hydrogeological conditions. The VDF identification was based on the factors D and I in DRASTIC and the contaminant migration and transformation parameters most closely associated with the vadose zone in the HYDRUS-1D model. Because the method is applicable to waters other than pore water with similar vadose zone structures, such as fracture water and karst water, it is extendible to a wide range of regions. The method can be adapted to fracture and karst waters by adding or removing VDFs.

Because the VDFs are identified and sequenced by a simulation process, the method is more objective than other methods, and can rationally predict the groundwater specific vulnerability at small-scale pollution sites. Furthermore, when combined with assessments of groundwater pollution intensity^3^, the method could ensure adequate protection of unconfined groundwater.

## Additional Information

**How to cite this article:** Li, J. *et al*. Identification of dominating factors affecting vadose zone vulnerability by a simulation method. *Sci. Rep.* **7**, 45955; doi: 10.1038/srep45955 (2017).

**Publisher's note:** Springer Nature remains neutral with regard to jurisdictional claims in published maps and institutional affiliations.

## Figures and Tables

**Figure 1:**
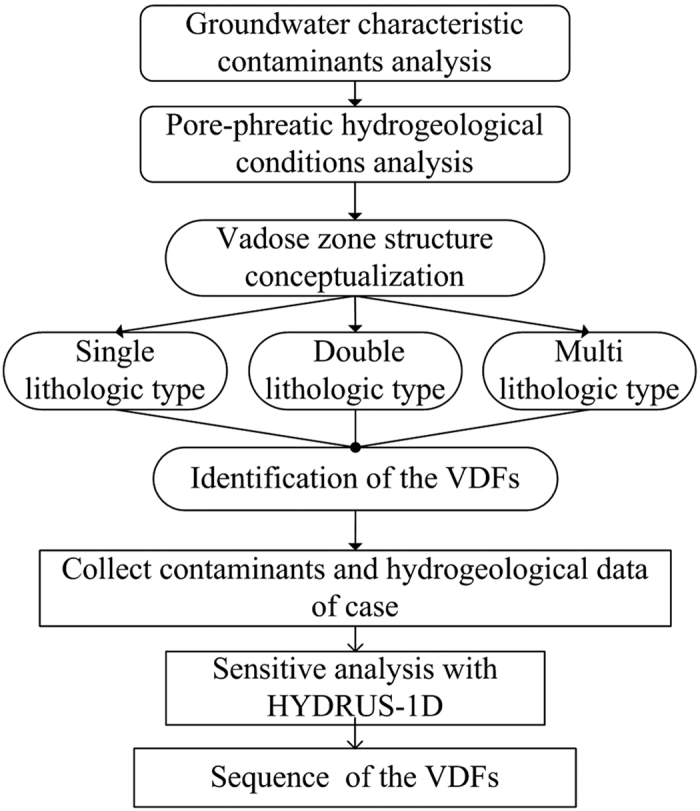
Identification and sequencing of the VDFs (generated by Microsoft Office 2010 software for Windows 2003/XP/VISTA/7).

**Table 1:** Empirical permeability coefficients in different media.

Media	Empirical permeability coefficient (cm/s)
Clay	<1.2 × 10^−6^
Silty clay	1.2 × 10^−6^~6.0 × 10^−5^
Silt	6.0 × 10^−5^~6.0 × 10^−4^
Silty sand	6.0 × 10^−4^~1.2 × 10^−3^
Fine sand	1.2 × 10^−3^~6.0 × 10^−3^
Medium sand	6.0 × 10^−3^~2.4 × 10^−2^
Coarse sand	2.4 × 10^−2^~6.0 × 10^−2^
Gravel	6.0 × 10^−2^~1.8 × 10^−1^

**Table 2:** VDFs of three typical vadose zone structures.

Vadose zone structure	VDFs	Factor number
Single lithologic type	M, K_1_ or K_2_, Kd, μ	4
Double lithologic type	M, M_1_, K_1_, K_2_, Kd, μ	6
Multi lithologic type	M, M_1_, 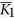 , 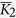 , Kd, μ	6

**Table 3:** Contaminants in the pollution source data of the case-study sites.

Pollution source identification	Site 1	Site 2	Site 3
Source location	Ground surface	In vadose zone	Ground surface
Source release mode	Continuous	Continuous	Continuous
Particular contaminant	Chromium	Ammonia nitrogen	Chlorobenzene
Initial concentration	400 mg/L	1810 mg/L	138 mg/L

**Table 4:** Results of the hydrogeological condition analysis.

Hydrogeological condition	Site 1	Site 2	Site 3
Annual precipitation	500 mm/a	600 mm/a	600 mm/a
Vadose zone structure	Single lithologic type	Double lithologic type	Multi lithologic type
VDFs	M, K, Kd, μ	M, M1, K1, K2, Kd, μ	M, M1, K1, K2, Kd, μ
Groundwater depth	4 m	25 m	9 m
Contaminants concentration in groundwater	55 mg/L	63.9 mg/L	5 mg/L

**Table 5:** Soil hydraulic and solute transport parameters.

Site	Soil hydraulic parameters	θr (−)	θs (−)	α (cm^−1^)	n (−)	Ks (cm/s)	L
Site 1	Silty clay	0.07	0.36	0.005	1.09	3.3 × 10^−5^	0.5
Site 2	Sand	0.045	0.43	0.120	1.89	1.2 × 10^−2^	0.5
	Gravel	0.057	0.46	0.124	2.28	4.0 × 10^−2^	0.5
Site 3	Silty clay	0.07	0.36	0.005	1.09	3.5 × 10^−5^	0.5
	Silt	0.095	0.41	0.019	1.31	7.5 × 10^−5^	0.5
**Site**	**Transport parameters**	**Bulk.density (g/cm**^**3**^)	**D**_**L**_**(cm)**	**D**_**w**_ **(cm/d**^**2**^)	**Kd (g/cm**^**3**^)	**μ (day**^**−1**^)	
Site 1	Silty clay	1.4	1.0	0.8	1	0.001	
Site 2	Sand	1.5	3.2	4	0.05	0.005	
	Gravel	1.6	3.5	4	0.03	0.004	
Site 3	Silty clay	1.4	1.0	0.9	1.0	0.001	
	Silt	1.43	2	0.9	0.05	0.0008	

**Table 6:** Simulation settings and calculation results.

Site	C_max_ (mg/L)	t (days)	C_0_ (mg/L)	T (days)	C_max_/C_0_ (−)	t/T (−)	n (−)
Site 1	64.19	10950	400	10950	0.16	1	0.16
Site 2	83.33	255	1810	7300	0.05	0.03	1.67
Site 3	19.51	3633	138	5475	0.14	0.66	0.21

**Table 7:** VDF parameters.

VDFs	M (m)	K (cm/s)	Kd (g/cm^3^)	μ (day^−1^)		
Site 1	4	3.3 × 10^−5^	1	0.001		
**VDFs**	**M (m)**	**M_1_ (m)**	**K_1_ (cm/s)**	**K_2_ (cm/s)**	**Kd (g/cm^3^)**	**μ (day^−1^)**
Site 2	25	2	1.2 × 10^−2^	4.0 × 10^−2^	0.03	0.004
Site 3	9	6	3.5 × 10^−5^	7.5 × 10^−5^	0.05	0.0008

**Table 8:** Vulnerability index amplitude |δ| at site 1.

VDFs amplitude		M	K	Kd	μ
original value	n_0_	0.16	0.16	0.16	0.16
Single VDF value increasing 20%	n’	0.061	0.162	0.115	0.112
Single VDF value decreasing 20%	n’	0.26	0.158	0.174	0.231
|δ|		0.62	0.01	0.37	0.18
Rank of VDFs		1	4	2	3

**Table 9:** Vulnerability index amplitude |δ| at site 2.

VDFs amplitude		M	M_1_	K_1_	K_2_	Kd	μ
original value	n_0_	1.67	1.67	1.67	1.67	1.67	1.67
Single VDF value increasing 20%	n’	1.28	1.55	1.674	1.672	1.64	1.42
Single VDF value decreasing 20%	n’	2.20	1.80	1.665	1.668	2.13	2.00
|δ|		0.27	0.075	0.002	0.001	0.15	0.17
Rank of VDFs		1	4	5	6	3	2

**Table 10:** Vulnerability index amplitude |δ| at site 3.

VDFs amplitude		M	M_1_	K_1_	K_2_	Kd	μ
original value	n_0_	0.21	0.21	0.21	0.21	0.21	0.21
Single VDF value increasing 20%	n’	0.13	0.2102	0.212	0.209	0.186	0.141
Single VDF value decreasing 20%	n’	0.39	0.2108	0.208	0.211	0.234	0.313
|δ|		0.632	0.003	0.008	0.004	0.113	0.408
Rank of VDFs		1	6	4	5	3	2
